# The rabbit as an animal model to study innate immunity genes: Is it better than mice?

**DOI:** 10.3389/fimmu.2022.981815

**Published:** 2022-09-09

**Authors:** João Soares, Ana Pinheiro, Pedro José Esteves

**Affiliations:** ^1^ Departamento de Ciências dos Computadores, Faculdade de Ciências, Universidade do Porto, Porto, Portugal; ^2^ Centro de Sistemas de Computação Avançada - Instituto de Engenharia de Sistemas e Computadores, Tecnologia e Ciência (CRACS - INESC TEC), Porto, Portugal; ^3^ CIBIO-UP, Centro de Investigação em Biodiversidade e Recursos Genéticos, Universidade do Porto, InBIO, Laboratrio Associado, Vairão, Portugal; ^4^ BIOPOLIS Program in Genomics, Biodiversity and Land Planning, CIBIO, Vairão, Portugal; ^5^ Departamento de Biologia, Faculdade de Ciências, Universidade do Porto, Porto, Portugal; ^6^ CITS - Centro de Investigação em Tecnologias de Saúde, Cooperativa de Ensino Superior Politécnico e Universitário (CESPU), Gandra, Portugal

**Keywords:** European rabbit, mouse, human, genetic distance, innate immunity genes

## Abstract

The European rabbit (*Oryctolagus cuniculus*) was the first animal model used to understand human diseases like rabies and syphilis. Nowadays, the rabbit is still used to study several human infectious diseases like syphilis, HIV and papillomavirus. However, due to several mainly practical reasons, it has been replaced as an animal model by mice (*Mus musculus*). The rabbit and mouse share a recent common ancestor and are classified in the superorder Glires which arose at approximately 82 million years ago (mya). These species diverged from the Primates’ ancestor at around 92 million years ago and, as such, one expects the rabbit-human and mouse-human genetic distances to be very similar. To evaluate this hypothesis, we developed a set of tools for automatic data extraction, sequence alignment and similarity study, and a web application for visualization of the resulting data. We aligned and calculated the genetic distances for 2793 innate immune system genes from human, rabbit and mouse using sequences available in the NCBI database. The obtained results show that the rabbit-human genetic distance is lower than the mouse-human genetic distance for 88% of these genes. Furthermore, when we considered only genes with a difference in genetic distance higher than 0.05, this figure increase to 93%. These results can be explained by the increase of the mutation rates in the mouse lineage suggested by some authors and clearly show that, at least looking to the genetic distance to human genes, the European rabbit is a better model to study innate immune system genes than the mouse.

## 1 Introduction

The purpose of developing animal models for scientific research is to establish an experimental system that can be reproduced in humans and or in veterinary research. The European rabbit (*Oryctolagus cuniculus*) was one of the first animal models used in immunological studies and was essential, for example, for the development of the rabies vaccine by Louis Pasteur in 1811 (1, 2). Furthermore, the study of rabbit immunoglobulins allowed us to understand much of what is known about the structure, function and expression of antibodies [reviewed in (3, 4)]. Anti-allotype antisera obtained in rabbit contributed to understanding of B cell development and revealed that individual B cells expressed only one of two alleles (allelic exclusion) [reviewed in (5)]. In addition, demonstration that genetic recombination occurred between immunoglobulin heavy chain variable regions (IGHV) and immunoglobulin heavy chain constant regions (IGHC) genes was an early indicator that immunoglobulins were encoded by more than one genetic unit (6).

Although the rabbit was one of the main animal models used in molecular immunology in the late 1980s, rabbits have been largely replaced by the house mouse (*Mus musculus*). The increase in the use of mice has found ground in their reduced maintenance costs, small size, ease of breeding with a short reproductive cycle and high number of progeny, wide availability of commercial immunological reagents and availability of inbred strains, knockouts (KO) and transgenic models (reviewed in (7)). The use of rabbits has, however, several advantages over the use of mice. Rabbits have a longer life span than mice and their size, bigger than mice, allows the sampling of blood and access to many cells and tissues from a single animal. Additionally, rabbits are reservoirs of several pathogens that cause zoonotic diseases. (reviewed in (7)). As such, the rabbit is still a reliable disease model for development of therapeutics and vaccines and studies of the cellular and molecular mechanisms underlying many human diseases, like syphilis, tuberculosis, HIV-AIDS, acute hepatic failure and diseases caused by noroviruses, poxvirus, herpes simplex virus, and papillomaviruses [reviewed in (7, 8)].

Furthermore, the rabbit is an excellent model to study the immune system evolution because it has unique features, like the use of mainly only one variable heavy chain (VH) gene in the antibody rearrangement (9) or having at least 15 immunoglobulin’s A (IgA) (10) with different hinge regions that show different resistances against bacterial proteases activity (11). These observations are amazing examples of immune system adaptations most likely driven by pathogen interaction. Despite these uniquenesses, in the innate immune system genes rabbit and other lagomorphs share with primates some peculiarities: a high selective pressure in the PRYSPRY domain of TRIM5 (12, 13), a gene conversion in the second extracellular loop of CCR5 (14, 15) and high mutation rate in the CD4 molecule (16). These are some examples that make the rabbit an excellent model to study human immune system genes.

Finally, rabbit and mouse are related species that share a recent common ancestor. Both species are grouped in the super order Glires which arose at approximately 82 million years ago (mya), but each is classified in a different order, the rabbit is a lagomorph and the mouse is a rodent (17). These species diverged from the Primates’ ancestor at around 92 million years ago (17) and, as such, one expects the rabbit-human and mouse-human genetic distances to be very similar. Interestingly, some evolutionary studies on immune system genes suggested that the genetic divergence between humans and rabbits was smaller than between mice and humans (18–20). These studies, however, focused on a very small number of immune system genes.

In this study, we tested the hypothesis that since the rabbit and mouse diverged from the human ancestor at the same time, they will have a similar genetic distance to human. To do so, we used a subset of the innate immune system gene repertoire of InnateDB [21] (https://www.innatedb.com/), a publicly available database of genes, proteins, experimentally-verified interactions and signaling pathways involved in the innate immune response to identify the largest number of innate immune system genes represented for humans and other species. From the subset of 4723 genes names (available in https://www.innatedb.com/redirect.do?go=resourcesGeneLists under the *Immport* label), we searched for available annotated sequences for the three species (i.e., human, mouse and rabbit), using NCBI’s Gene and Nuccore databases (https://www.ncbi.nlm.nih.gov/), resulting in a set of 3580 mRNA and CDS sequences. Finally, for each gene we compared the rabbit-human and mouse-human genetic distances based on the aligned CDS sequences for orthologous genes.

## 2 Materials and methods

For this study we developed a set of tools for automatic data extraction, sequence alignment and similarity study, and a web application for visualization of the resulting data.

### 2.1 Data extraction

**Figure 1:**
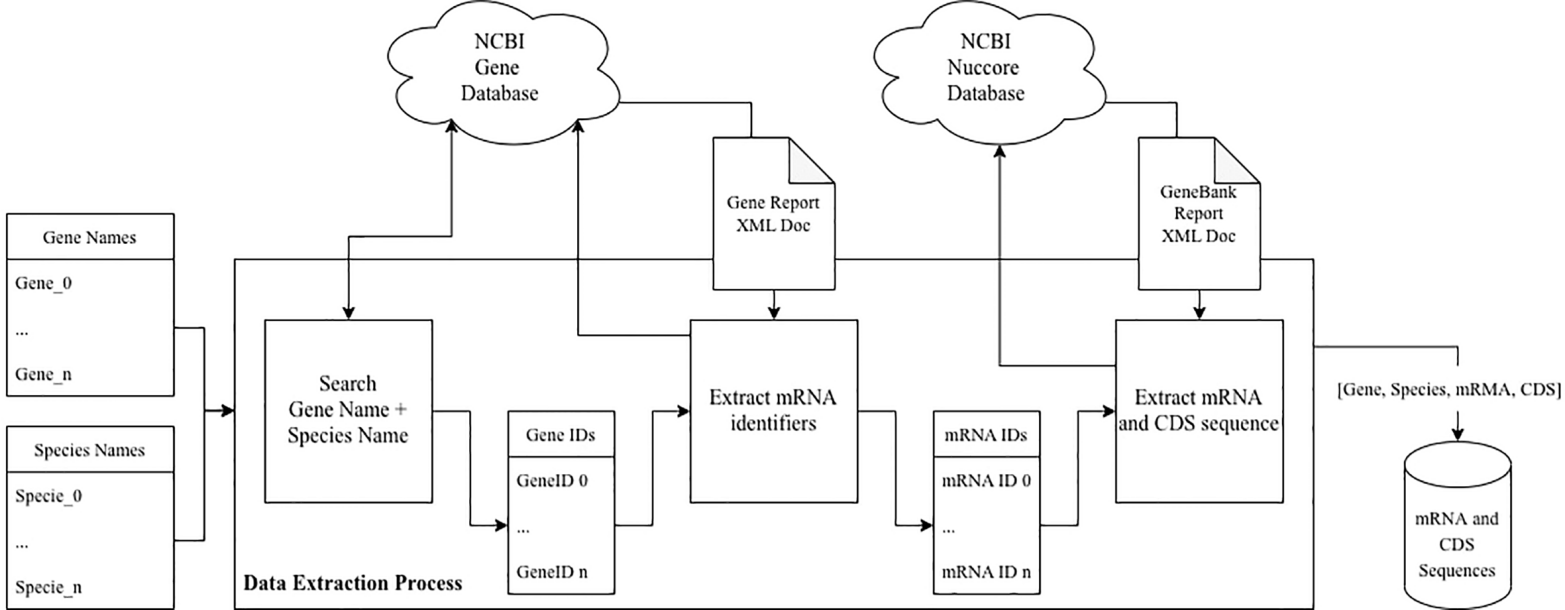
Data extraction pipeline.

For the data extraction process we designed a tool that, given a set of gene names and a set of species names downloads the mRNA gene sequences and corresponding CDS sequences of known resulting products available in the NCBI’s Annotated Genomes, for each gene and species combination, as presented in **Figure 1**. In cases where more than one resulting product is available (i.e., multiple mRNA and CDS sequences for the same gene of the same species), the X1 isoforms were selected.

This tool was implemented in Python and the NCBI database was used as a data source (using the Entrez Programming Utilities (E-utilities) API). For each gene name and species name combination, a gene search was conducted (on the Gene NCBI database), using the corresponding URL and the respective arguments. This returned the gene identifiers of the different genes. For each gene identifier, the corresponding gene report was downloaded in XML format (again, using the Gene NCBI database) and parsed to extract the set of mRNA Reference Sequences (RefSeq) identifiers.

For each RefSeq identifier, the corresponding GenBank data was downloaded, in XML format (using the Nuccore NCBI database). From the resulting data the mRNA sequence and the corresponding CDS sequences were extracted.

### 2.2 Alignment and similarity

**Figure 2:**
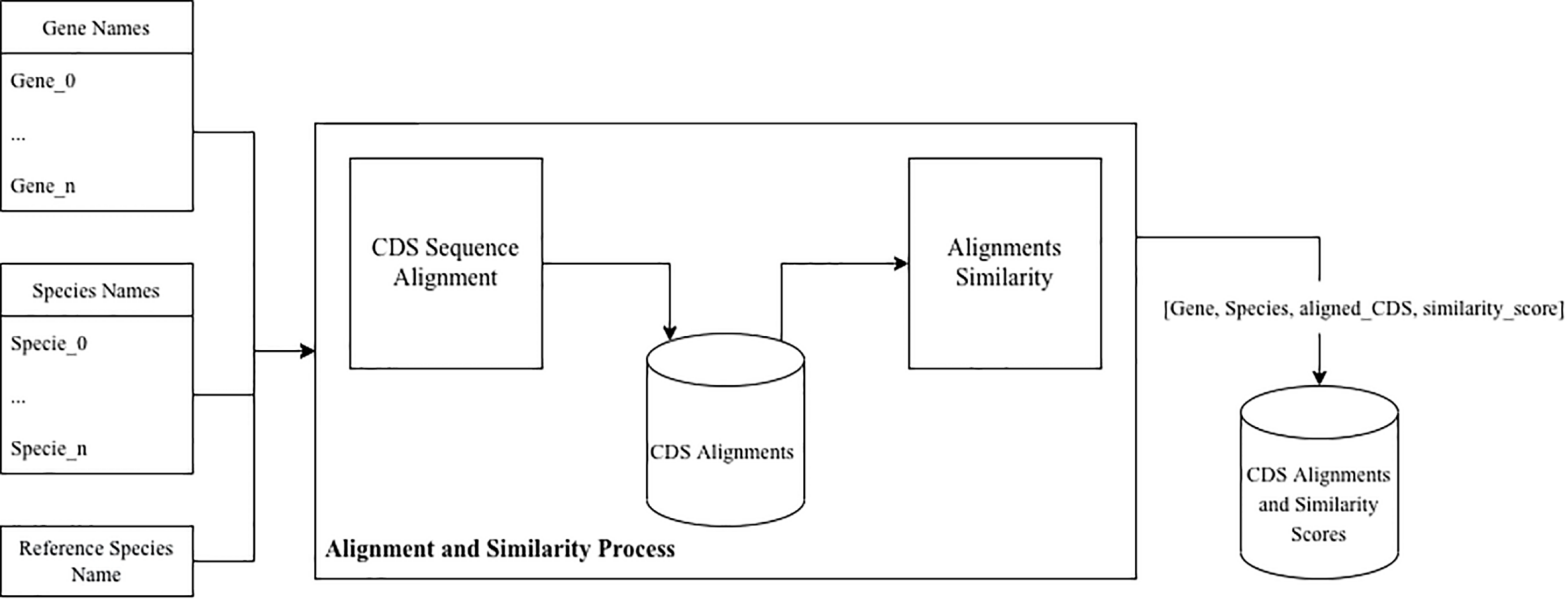
Data processing pipeline.

For the sequence alignment and similarity process we designed a tool that, given a set of gene names, a set of species names, and reference species name aligns the CDS sequences and measures their similarity against the given reference, as presented in **Figure 2**. This tool was implemented in Python, and uses the data resulting from the Data Extraction process as input.

For each gene name, the CDS sequences from each species (including reference species) was selected. The sequences are then aligned against the reference sequence, using Python’s Biopython library. For the alignment, a local pairwise alignment strategy was selected using the match score and gap penalty parameters similar to the ones used in NCBI’s Blast tool (i.e., 2, -3, -5, -2 for matching, mismatching, gap opening and gap extension respectively). The similarity score was calculated by counting the number of equal base pairs and dividing it by the difference between the length and the number of indels of the aligned sequence.

The final relative similarity data set was constructed by measuring, for each gene, the difference between the similarity score for the different species against the reference one.

### 2.3 Visualization

For the visualization process we designed a web application for visualizing the similarity scores obtained from the Alignment and Similarity process. This was implemented in HTML and javascript, using NodeJS and Express for the back-end Web service, and HTML, Bootstrap and Chart.js for the front-end.

The application (available in https://warm-plains-91813.herokuapp.com/) allows the user to visualize the relative similarity from the set of genes common among the different species. Additionally, it allows users to select specific genes and visualize the respective information (including Gene Bank and Nuccore data) as well as the similarity results and resulting alignments.

### 2.4 Workflow

**Figure 3:**
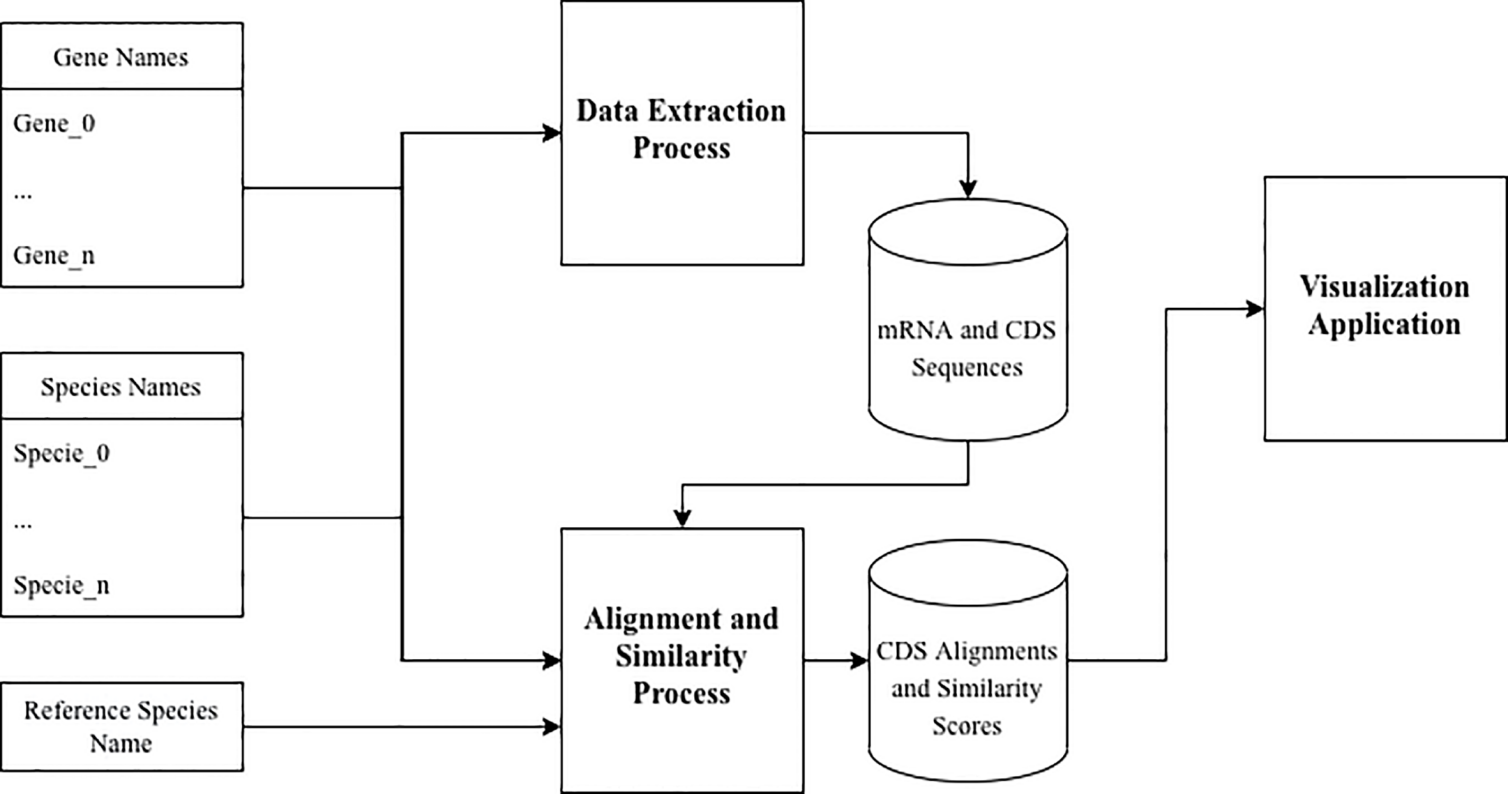
Process workflow.

We now describe the workflow for the data extraction, processing and visualizing processes used in our study, as presented in **Figure 3**.

The set of gene names [available in InnateDB (21)] and the set of species names (*Homo sapiens*, *Mus musculus* and *Oryctolagus cuniculus*) was used as input to the Data Extraction pipeline. The resulting data from this process was a dataset of gene names, species names, mRNA and CDS sequences available for each orthologous gene of the three species.

This dataset, in conjunction with the set of gene names, the set of species names and the reference species name was then used as input to the Alignment and Similarity process. The resulting data from this process was a dataset of gene names, species names, aligned CDS sequences and similarity scores between the aligned sequences.

The visualization process uses the dataset produced by the Alignment and Similarity process from calculating and displaying the relative similarities for each orthologous gene of the three species.

### 2.5 Genetic comparison of some key immune system genes between human, rabbit and mouse

The phylogenetic relationships and genetic distances, nucleotide and amino acid, between human, rabbit and mouse were obtained for four key innate immune system genes: CD4, ABCB11, IL2 and MYO1E. The full CDS sequences for these genes were obtained from Genbank (accession numbers for the sequebces used are given in **Figure 5**) and aligned using CLUSTAL W (22) as implemented in BioEdit v7.2.5 (23), and corrected manually as necessary. MEGA version X software (24) was used to construct a Maximum likelihood (ML) phylogenetic tree and to calculate genetic distances. The phylogenetic tree was constructed using the GTR model of nucleotide substitution. This software was also used to calculate the nucleotide and amino acid distances using the p-distance method and pairwise deletion of gaps options.

## 3 Results obtained comparing the genetic differences between human-mouse and human-rabbit

From the 3580 gene sequences studied, we selected sequence alignments with more than 150 bp resulting in 2793 genes. The rational for this selection was to avoid partial match sequences or small matches that can happen by chance (without biological meaning).

**Figure 4:**
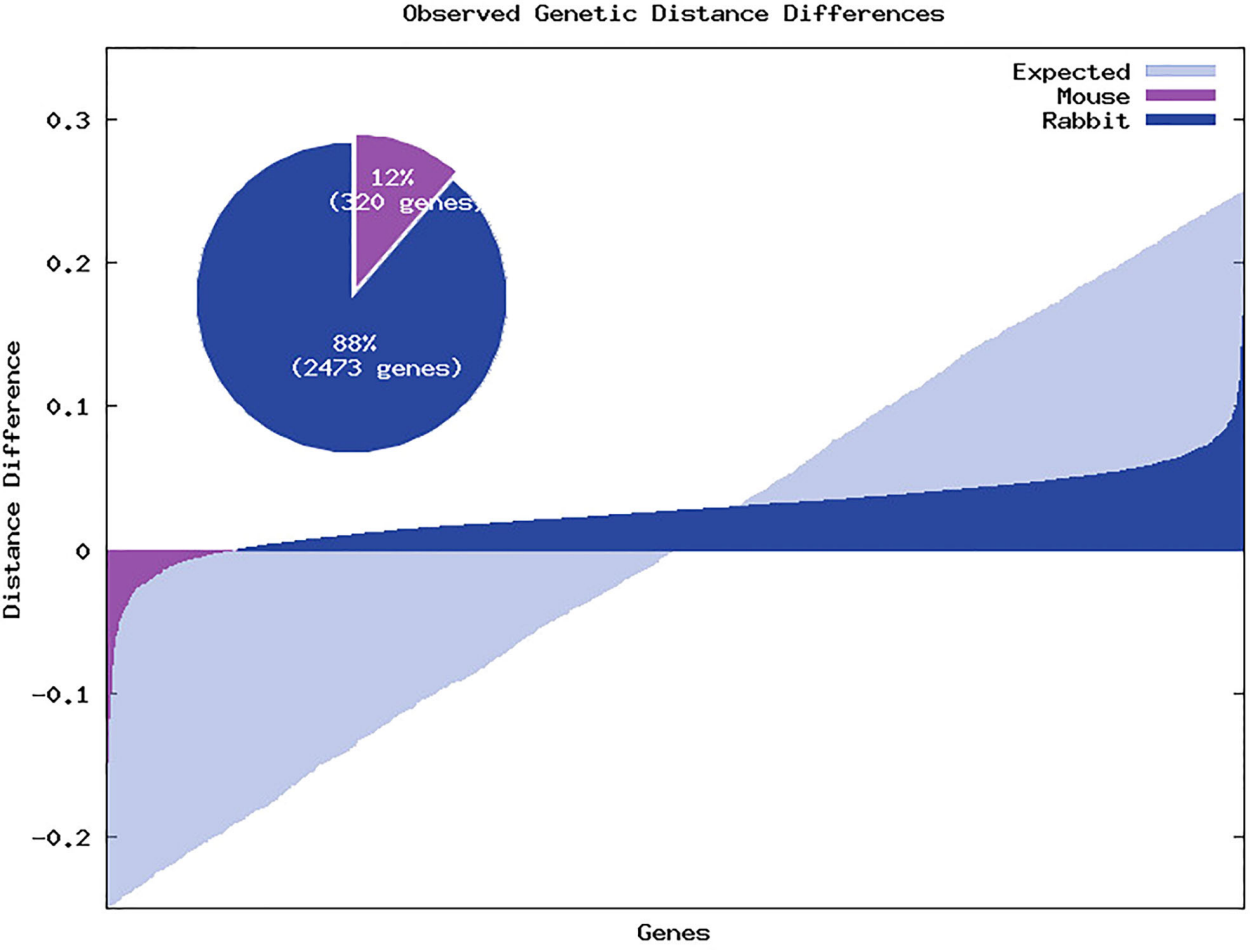
Genetic distance differences between rabbit and human and mouse and human. Positive values represent genes in which the genetic distances between mouse and human is greater than the genetic distances between rabbit and human. Light blue regions represent the expected relative gene distances between the similarities between rabbit and human and mouse and human. Dark blue regions represent the relative distances in which gene similarity between rabbit and human is higher than between mouse and human. Violet regions represent the relative distances in which gene similarity between rabbit and human is lower than between mouse and human. The pie chart represents the percentage of genes for which the genetic distances between rabbit and human are lower than mouse and human (in dark blue) and for which the genetic distances between mouse and human are lower than rabbit and human (in violet).

**Figure 5:**
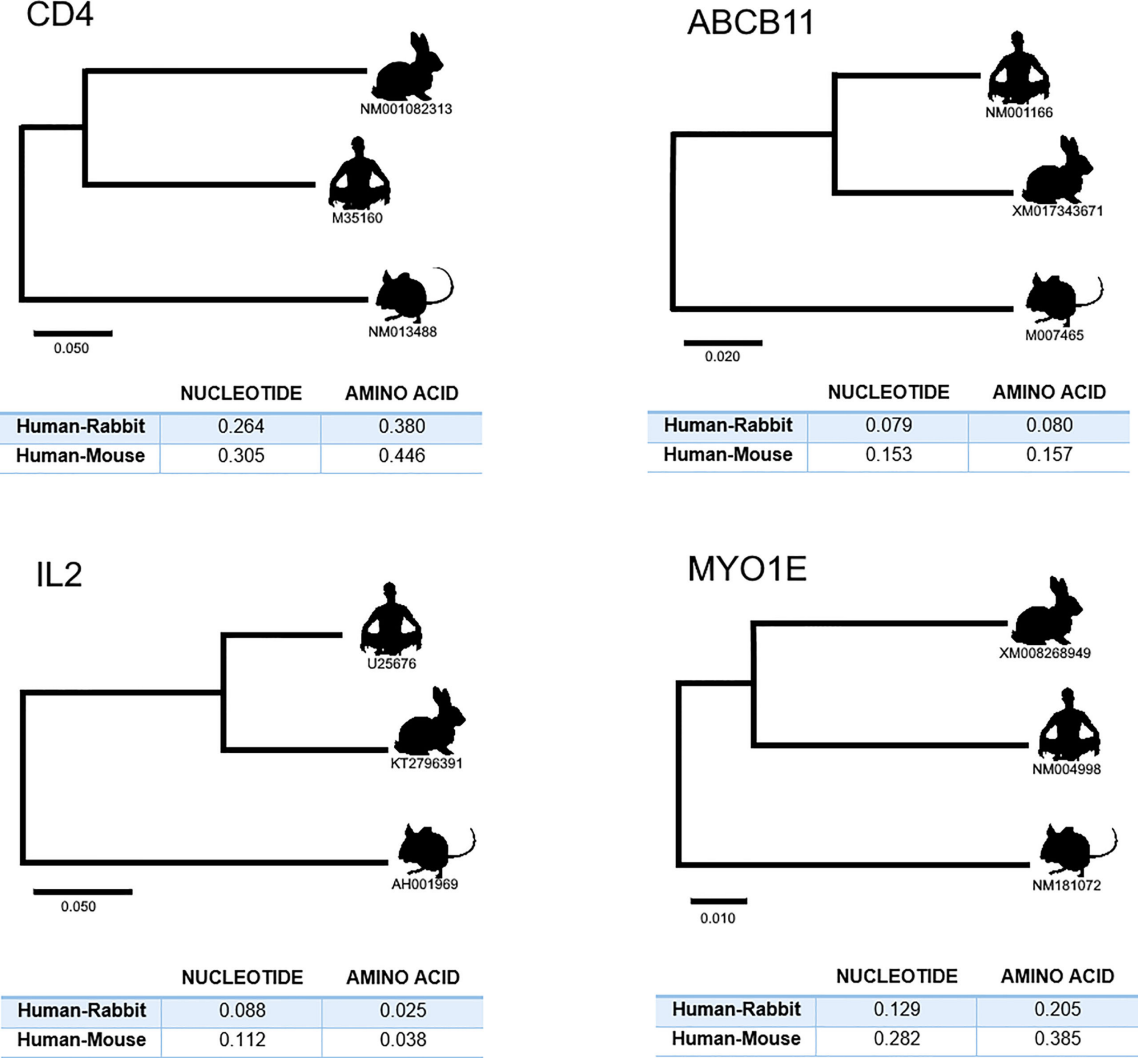
Phylogenetic relationships and genetic distances (nucleotide and amino acid) between human, rabbit and mouse for four key innate immune system genes: CD4, ABCB11, IL2 and MYO1E. Maximum likelihood (ML) method and the GTR model of nucleotide substitution were used to obtain each gene phylogenetic tree. Accession numbers of the used sequences are shown under the species symbol in the trees. The genetic distances are shown in the tables. These were obtained using the options p-distance and pairwise deletion.

From the 2793 genes, 320 (12%) showed a genetic distance between mouse and human lower than the obtained between rabbit and human, 2468 (88%) showed a genetic distance between mouse and human higher than the obtained between rabbit and human and in 5 genes we observed identical genetic distance between mouse and human and rabbit and human (see **Figure 4**). Furthermore, when we selected only genes that showed at least 0.05 of difference between the mouse-human and rabbit-human distances, the ratio increased to 429 (93%) genes showing a genetic distance between mouse and human higher than the obtained between rabbit and human and only 30 genes (7%) showing a genetic distance between mouse and human lower than the obtained between rabbit and human, despite the decrease in the number of genes to 460. To further explore the differences between the human-mouse and human-rabbit genetic distances we selected some key innate immune system genes and using the complete CDS we compared the genetic similarity between these species using phylogenetic trees and nucleotide and amino acid distances. The four selected genes were: 1) CD4 that acts as a coreceptor with the T-cell receptor on the T lymphocyte to recognize antigens and it is also a primary receptor for entry of the HIV; 2) Interleukin-2 (IL2) that encodes a secreted cytokine produced by activated CD4+ and CD8+ T lymphocytes, that is important for the proliferation of T and B lymphocytes; 3) ATP binding cassette subfamily B member 11 (ABCB11) that transport various molecules across extra- and intra-cellular membranes. It is a member of the MDR/TAP subfamily that are involved in multidrug resistance. The protein encoded by this gene is the major canalicular bile salt export pump in man; and 4) myosin IE (MYO1E) encodes a member of the nonmuscle class I myosins which are a subgroup of the unconventional myosin protein family. This protein localizes to the cytoplasm and may be involved in intracellular movement and membrane trafficking. For each gene, both nucleotide and amino acid genetic distances were smaller between rabbit and human than mouse and human, as presented in **Figure 5**.

## 4 Discussion

The increase in the number and quality of entire genomes in the last years allows us to get a better picture of the number and the variability of genes between species. Taking advantage of the high-quality genomes available for human, rabbit and mouse, we tested our hypothesis that since the rabbit and mouse shared a recent common ancestor and diverged from human ancestor at the same time, they will have a similar genetic distance to human. To test this hypothesis we compared the genetic variability of 2793 innate immunity genes.

The results obtained showed that the genetic diversity between rabbit and human is clearly lower than that between mouse and human. These results are in line with previous observation obtained with some immune genes (18–20). The most likely explanation for the observed difference in the mouse-human and rabbit-human genetic distances is the increase of the mutation rate in the rodent lineage that has already been suggested by other authors [e.g (25, 26)]. The obtained results clearly show that, at least looking at the genetic distance to human genes, the European rabbit is a better model to study innate immune system genes that the mouse.

## Data availability statement

Publicly available datasets were analyzed in this study. This data can be found here: https://warm-plains-91813.herokuapp.com/.

## Author contributions

JS designed and developed the tools for data extraction, analysis and data visualization. AP and PE participated in the designing process and discussed the data. PE conceived the study. All authors contributed to the article and approved the submitted version.

## Funding

This work was funded by the project NORTE-01-0246-FEDER-000063, supported by Norte Portugal Regional Operational Programme (NORTE2020), under the PORTUGAL 2020 Partnership Agreement, through the European Regional Development Fund (ERDF). FCT also supported the post-doctoral fellowships of AP (SFRH/BPD/117451/2016) and the Investigator grant of PE (CEECIND/CP1601/CT0005).

## Conflict of interest

The authors declare that the research was conducted in the absence of any commercial or financial relationships that could be construed as a potential conflict of interest.

## Publisher’s note

All claims expressed in this article are solely those of the authors and do not necessarily represent those of their affiliated organizations, or those of the publisher, the editors and the reviewers. Any product that may be evaluated in this article, or claim that may be made by its manufacturer, is not guaranteed or endorsed by the publisher.
